# EXPERIENCES AND WELL-BEING OF LOW-INCOME WORKERS IN THE SENIOR COMMUNITY SERVICE EMPLOYMENT PROGRAM

**DOI:** 10.1093/geroni/igad104.0887

**Published:** 2023-12-21

**Authors:** Cal Halvorsen, Patrick Lai, Elizabeth Howard, Karen Lyons, Sara Moorman, Christina Matz

**Affiliations:** Boston College, Boston, Massachusetts, United States; Boston College School of Social Work, Boston, Massachusetts, United States; Boston College Connell School of Nursing, Boston, Massachusetts, United States; Boston College, Boston, Massachusetts, United States; Boston College, Boston, Massachusetts, United States; Boston College, Boston, Massachusetts, United States

## Abstract

The Senior Community Service Employment Program (SCSEP) is the only federal job-training program specifically for older workers. SCSEP enrolls unemployed older workers with incomes at or below 125% of the federal poverty level into on-the-job and classroom-based training. SCSEP participants often struggle to secure unsubsidized work, as they experience, on average, more than 3 barriers to employment (e.g., low literacy levels, disability, limited English proficiency). While some outcome metrics are tracked (e.g., job attainment, median unsubsidized wages), we know relatively little about participants’ health, well-being, and training experiences. In response, and in partnership with the Massachusetts Executive Office of Elder Affairs, we fielded a survey on the multidimensional health, well-being, and experiences of participants throughout Massachusetts. Fielded in the spring and summer of 2022 and in six languages, a total of 91 SCSEP participants took the survey with an age range of 57 to 82. Almost half (44%) spoke a language other than English at home, of which Cantonese and Vietnamese were the most common. Respondents generally reported being in moderately good health (only 3% reported “excellent” health) with at least one chronic health condition (86%), and two-thirds (66%) reported reduced social activities due to the COVID-19 pandemic. Less than one-quarter (22%) reported having money left over at the end of the month, but that SCSEP itself improved their finances, social engagement, and self-confidence. To conclude, we will offer reflections on the importance of tracking additional characteristics of SCSEP participants and engaging in respectful community-based work with diverse older populations.

